# The Use of Artificial Intelligence in the Liver Histopathology Field: A Systematic Review

**DOI:** 10.3390/diagnostics14040388

**Published:** 2024-02-10

**Authors:** Flavia Grignaffini, Francesco Barbuto, Maurizio Troiano, Lorenzo Piazzo, Patrizio Simeoni, Fabio Mangini, Cristiano De Stefanis, Andrea Onetti Muda, Fabrizio Frezza, Anna Alisi

**Affiliations:** 1 Department of Information Engineering, Electronics and Telecommunications (DIET), “La Sapienza”, University of Rome, 00184 Rome, Italy; flavia.grignaffini@uniroma1.it (F.G.); francesco.barbuto@gmail.com (F.B.); lorenzo.piazzo@uniroma1.it (L.P.); fabio.mangini@uniroma1.it (F.M.); fabrizio.frezza@uniroma1.it (F.F.); 2 Research Unit of Genetics of Complex Phenotypes, Bambino Gesù Children’s Hospital, IRCCS, 00165 Rome, Italy; maurizio.troiano@opbg.net (M.T.); cristiano.destefanis@opbg.net (C.D.S.); 3 National Transport Authority (NTA), D02 WT20 Dublin, Ireland; patrizio.simeoni@nationaltransport.ie; 4 Faculty of Lifelong Learning, South East Technological University (SETU), R93 V960 Carlow, Ireland; 5 Bambino Gesù Children’s Hospital, IRCCS, 00165 Rome, Italy; andrea.onettimuda@opbg.net

**Keywords:** liver, biopsy, histology, histological images, computer-aided diagnostics, artificial intelligence, machine learning, deep learning, convolutional neural networks

## Abstract

Digital pathology (DP) has begun to play a key role in the evaluation of liver specimens. Recent studies have shown that a workflow that combines DP and artificial intelligence (AI) applied to histopathology has potential value in supporting the diagnosis, treatment evaluation, and prognosis prediction of liver diseases. Here, we provide a systematic review of the use of this workflow in the field of hepatology. Based on the PRISMA 2020 criteria, a search of the PubMed, SCOPUS, and Embase electronic databases was conducted, applying inclusion/exclusion filters. The articles were evaluated by two independent reviewers, who extracted the specifications and objectives of each study, the AI tools used, and the results obtained. From the 266 initial records identified, 25 eligible studies were selected, mainly conducted on human liver tissues. Most of the studies were performed using whole-slide imaging systems for imaging acquisition and applying different machine learning and deep learning methods for image pre-processing, segmentation, feature extractions, and classification. Of note, most of the studies selected demonstrated good performance as classifiers of liver histological images compared to pathologist annotations. Promising results to date bode well for the not-too-distant inclusion of these techniques in clinical practice.

## 1. Introduction

### 1.1. Artificial Intelligence in Medicine

Artificial intelligence (AI) refers to the ability of machines to perform tasks that typically require human intelligence, such as planning, problem-solving, natural language understanding, and learning. Although AI was born around the 1950s, it has only become very successful in the last two decades due to advances in technology, the development of new algorithms, and the availability of big data. In recent years, AI has experienced a rapid development in medicine. Besides medical research, the main applications of AI in medicine include health plan analysis, medical data management, digital consultation [1], the development of personalized treatment plans [2], and the automatic identification of diagnostic and predictive patterns [3]. Computer vision (CV), a subfield of AI concerned with extracting, interpreting, and understanding visual information from the world around us [4], enables the pursuit of these goals from medical images. In fact, through CV, images produced using X-rays [5,6,7], ultrasounds [8,9,10,11], mammograms [12,13,14,15], computed tomography (CT) scans [16,17,18,19], magnetic resonance imagining (MRI) scans [20,21,22], and dermoscopy [23,24,25,26,27], can be automatically analyzed to detect medical abnormalities and support a diagnosis.

### 1.2. AI Applications on Histological Images

**Figure 1:**
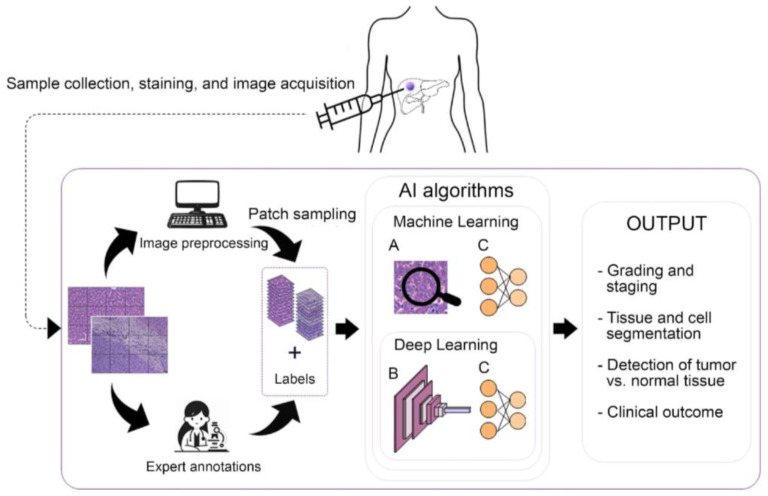
Principal CAD system steps for histopathological image inspection. (**A**) Manual features extraction. (**B**) Automatic feature extraction. (**C**) Classification with ML and DL.

In addition to traditional imaging, histological imaging has also been subjected to AI analysis to aid in detecting and diagnosing various diseases. Histological images are microscopic images of tissue samples taken by biopsy, an important tool for diagnosing and treating various medical conditions, including cancer, infection, and inflammatory diseases. In cancer pathology, AI-assisted diagnostic flowcharts in oncology fulfill several tasks, including the identification or segmentation of the region of interest (ROI) as a tumor region using whole-slide image (WSI) systems [28,29]; immunostaining scoring [30]; mitosis detection [31,32]; subtyping; classification [33,34,35,36,37,38,39,40,41,42,43,44]; staging and prognostic prediction; and identification of pathological features that could be used as biomarkers [45]. By allowing physicians to examine the tissue sample under a microscope and identify the presence of abnormal cells or tissues, a biopsy examination is necessary to provide an accurate diagnosis and monitor the effectiveness of treatment over time. However, biopsy analysis is a time-consuming procedure that is highly subjected to experience and inter- and intra-operator variability [46]. These limitations have necessitated the development of computer-assisted diagnostic (CAD) systems that leverage AI algorithms to identify and classify different cell types automatically, thus favoring the emergence of a pathology sub-field under the umbrella term of digital pathology (DP) [47]. The main steps of CAD systems for inspecting histopathological images (Figure 1) include sampling local mini patches from large WSIs, image pre-processing, feature extraction, and application of AI [48,49].

Many AI algorithms are used today in various fields, including rule-based AI, natural language processing (NLP), and artificial neural networks (ANNs). ANNs are typically divided into two subcategories, i.e., machine learning (ML) and deep learning (DL), which in turn represent the two most widely employed branches of AI nowadays. Both can be supervised or unsupervised, but the DL needs to have large amounts of data for the training phase (the training set), which is critical in many application fields, such as medicine.

The size of the training set is not always sufficiently large to provide a good convergence of the DL algorithm. In this case, especially in the field of CV, an operation that often proves crucial for improving performance, in particular for convolutional neural networks (CNN), is that of data augmentation (DA). DA consists of the creation of new additional artificial examples that are added to the training data, resulting in a larger training set [50,51,52]. Among the classic operations used for DA are image rotation, zooming, cropping, noise addition, scaling, and translation [53]. In addition to these standard operations, more advanced transformations, such as changing contrast or brightness, can be applied. In situations where the classes in the dataset are strongly unbalanced, the technique of DA is also used to balance the data in the classes by applying a higher augmentation for the fewer classes.

### 1.3. Aim

Recently, CV has shown promise in supporting the diagnosis and treatment of liver disease [54]. CV algorithms can be used to detect and segment liver lesions, such as tumors or cysts [55,56], to analyze liver texture and structure to identify areas of fibrosis or cirrhosis [57,58,59], and to develop models that can predict liver disease progression [60,61,62,63,64,65,66].

The present systematic review aims to provide a comprehensive overview of the ten-year literature evidence on AI applications to liver histopathology and explain their performance. In particular, here we summarize all the studies where AI was applied to histological imaging in the context of liver diseases in animals and humans. Finally, we discuss the contents of the selected articles and outline the main critical issues in the application of AI in histopathology, providing some suggestions that could be implemented for the application of DP in liver diseases.

## 2. Methods

In this systematic review, study inclusion and data extraction were performed in agreement with the Preferred Reporting Items for Systematic Reviews and Meta-Analysis (PRISMA) guidelines [67].

### 2.1. Search Strategy

A systematic search of the PubMed, SCOPUS, and Embase electronic databases was conducted between 1 January 2013 and 22 November 2023 to identify all articles concerning the use of ML and DL in the histopathology of liver diseases. The following keywords were used: (imaging) AND (microscopy) AND (Artificial Intelligence OR Machine Learning OR Deep Learning) AND (liver disease) AND (histology OR biopsy). A manual selection of relevant articles through crosschecking references was additionally performed.

### 2.2. Search Criteria

Original articles published within the past 10 years were included, limiting the language of publication to English and narrowing the type of study to case reports, classical original articles, clinical studies, meta-analyses, multicenter studies, and observational studies. The exclusion criteria regarded studies conducted on non-histopathological images (e.g., X-rays, ultrasounds, and MRI images) or non-WSIs (e.g., cell images), molecular mechanism analysis (e.g., omic data), and other diseases.

### 2.3. Screening and Article Selection

The examination and screening of all the search results were conducted by two independent authors (F.G. and A.A.). Articles were screened by title and abstract to identify those relevant to the topic covered in this review and based on previously introduced criteria. All duplicate and review articles were identified and eliminated. Subsequently, a careful reading and analysis of the remaining articles was conducted and resulted in determining the manuscripts discussed in the current revision.

### 2.4. Data Extraction

Data were collected and extracted from the articles selected by highlighting the following key aspects: AI tools used, study objective, number of human patients, type of animal models, DA techniques, main study results, and performance. The metrics for performance evaluation included data of accuracy (ACC), sensitivity (SE), specificity (SP), precision (PR), recall (REC), F1 score (F1), area under receiver operating characteristic (ROC) curve (AUC), Matthews’ correlation coefficient (MCC), Intersection over Union (IoU), Jaccard index, concordance index, coefficient of determination (R^2^), and Spearman’s correlation coefficient (r). The abbreviation “Av” indicates the average results over several repetitions of the experiment.

## 3. Results

### 3.1. Search Results

**Figure 2:**
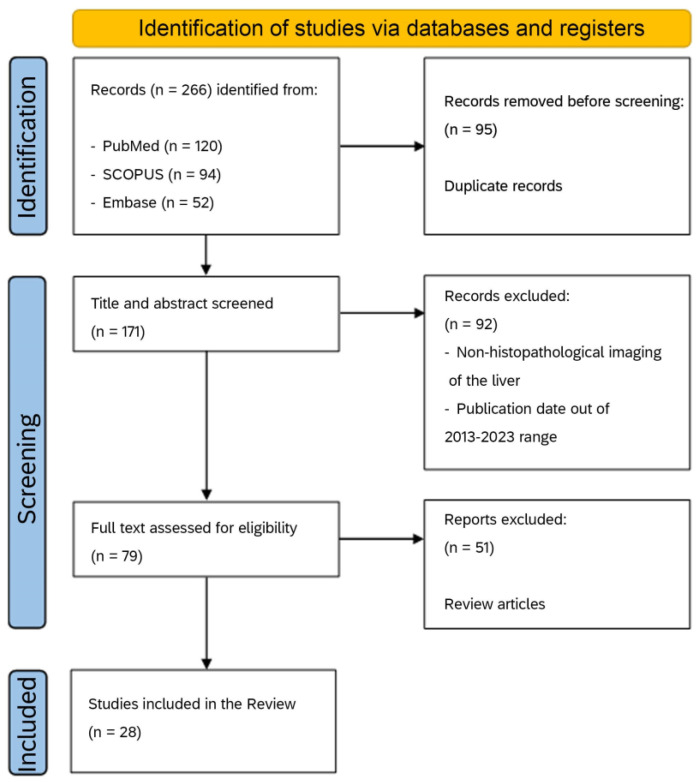
PRISMA flow diagram of article identification, exclusion, and number of the final articles discussed in the present review.

This section summarizes articles on the use of AI applied to liver histopathology collected from the literature after a careful search. Data extraction was performed following the PRISMA guidelines [67] (Figure 2). The initial search of electronic databases generated 266 articles (120 PubMed, 94 SCOPUS, and 52 Embase).

Among these articles, 95 were eliminated because they were duplicated records. After reading the title and abstract, 92 articles were discarded because they were outside the topic of the present review, following the exclusion criteria described above. During the next step, articles were carefully selected by reading the remaining 79 articles. After the removal of review articles, only 28 were deemed suitable for a complete evaluation of reported results.

### 3.2. Results Organization

**Figure 3:**
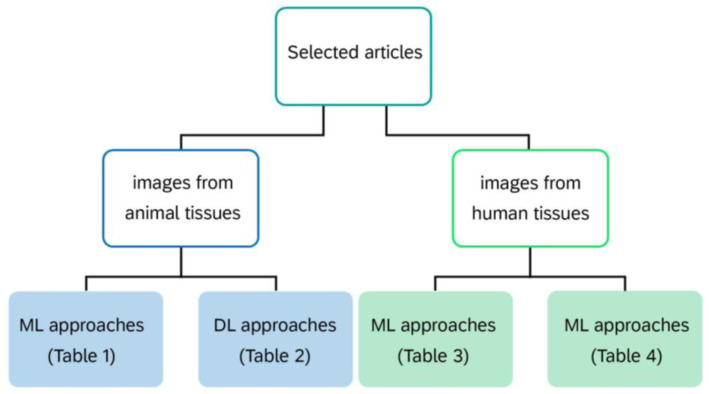
Flow diagram of results organization and presentation along the present review.

All selected articles were finally discussed by dividing them into two macro areas, depending on whether the research was conducted on animal (11 articles) or human (17 articles) tissue. Next, ML- and DL-based articles in each macroarea were included in a dedicated microarea (Figure 3). All studies in each microarea were discussed separately and summarized in different tables in chronological order. In particular, we organized the tables and review by discussing the studies related to animals in the first two tables, for ML and DL approaches, respectively; while those related to humans in the next two tables, for ML and DL approaches, respectively.

### 3.3. Studies Conducted on Animal Tissues

Our selection highlighted 11 articles on the application of AI approaches in animal models of liver diseases, including non-alcoholic fatty liver disease (NAFLD), drug-induced liver injury (DILI), and hepatic fibrosis. Table 1 and Table 2 summarize the studies conducted on animal tissues by distinguishing between those exploiting ML and DL approaches.

#### 3.3.1. ML Approaches on Animal Tissues

Intending to perform accurate quantification of hepatic steatosis, Homeyer et al. [68] presented a new method for the detection of fat droplets in histological images of the rat liver sections with mild, moderate, or severe steatosis from rats treated with a methionine-choline-deficient plus high-fat diet (HFD) between 3 days and 6 weeks. The authors proposed the use of adjacency statistics (i.e., simple statistics on neighboring pixels capturing texture features) as a tool for detecting shape features, thus avoiding the use of roundness as a unique criterion for fat droplet classification in steatotic livers. The proposed method involved two steps: the first step classified image pixels into background or tissue based on brightness and saturation values; the second step classified background blobs as fat droplets or other blanks based on adjacency statistics using a random forest (RF) classifier consisting of an ensemble of 20 decision trees. The authors used examples of fat drops and blanks annotated in 32 rat liver sections stained in hematoxylin and eosin (H&E) with mild, moderate, or severe steatosis. The metrics used to evaluate the method were SP, ACC, and SE. The last one was evaluated in separated fat drops (SFD) and clustered fat drops (CFD). Compared with the results obtained using classical shape features, such as size and eccentricity, or the combination of these with adjacency statistics, those obtained using adjacency statistics alone as shape features were significantly better, reaching a value of ACC of 91%.

The hepatic fibrosis staging was explored via staining using the Masson–Goldner method of normal murine livers and in livers from mice after carbon tetrachloride (CCl4) treatment for 2 and 4 weeks [69]. This approach, which stained nuclei, cytoplasm, and connective tissues with different colors (dark brown, red, and blue, respectively), was combined with a quantitative analysis of the spectral–spatial characteristics of multiphoton excitation fluorescence. Indeed, liver tissues were acquired via multiphoton excitation fluorescence of unstained or stained images, which then were segmented using spectral markers selected by combining a local morphological feature extraction algorithm and a k-means clustering method. Since this approach suggested that morphological features of individual segmented objects could characterize the liver fibrosis stage, the authors employed an ML approach called the bag-of-features framework for image classification: this included training and testing steps and classification based on a support vector machine (SVM). In this study, it was shown that local spectra of fibrillar collagen deposits correlate well with conventional scoring based on histologic staining specimens. Furthermore, spectral imaging of native emission fluorescence from liver tissue was shown to have the ability to differentiate not only between normal and diseased liver but also between the early stages of fibrosis and progressive disease states. This laid the foundation for spectroscopy-based ML of chronic liver diseases and could also be applied to a range of hepatopathies associated with autofluorescence alterations.

Yarbakth et al. [70] used a multimodal imaging approach to perform automatic identification of early septic liver damage in a mouse model of polymicrobial abdominal infection (PCI). For this purpose, six multimodal images, including coherent anti-Stokes Raman scattering (CARS), two-photon excitation fluorescence (TPEF), and second harmonic generation (SHG) for each animal in the control or PCI group, were acquired. Nine texture features were calculated for each image, for a total of 27 features, and then the median of the texture feature values was calculated for each mouse. After a reduction in the dimensionality of data, five principal components were used to train four binary linear discriminant analysis (LDA) classifiers to distinguish PCI and control groups. The authors highlighted that the classification performance via AUC obtained via the SHG channel was 49%, while the classification via TPEF and CARS channels was above 80% and 90%, respectively. The classifier based on the combination of all channels exhibited 85% AUC.

Wang et al. [71] described a system to quantify steatosis and fibrosis progression in animal models of diet-induced NAFLD using AI approaches on SHG/TPEF images. In the study, features were extracted from the WSIs for steatosis and fibrosis using an ML approach based on the classification and regression trees method. Even though the pipeline for feature extraction was not well explained, the study reported good performance of an AI approach to monitoring different types of steatosis and fibrosis in the NAFLD model.

#### 3.3.2. DL Approaches on Animal Tissues

For the detection and classification of DILI on histopathological liver biopsy images, a preclinical diagnostic support system was designed and proposed [72]. This study was the first attempt to classify DILI injury patterns based on WSIs. Two heterogeneity evaluation models were proposed: a feature model used to calculate fractals and lacunarity values and one based on DL. The latter exploits Google’s AutoML Visionbeta, an easy-to-use tool for non-experts in ML, thus allowing them to divide the dataset automatically into the training and test sets and choose both the model and its parameters. Histopathological images of liver tissues from rats with DILI induced by 10 different drugs stained with H&E were taken from the open-source toxicogenomics database “Toxicogenomics Project-Genomics Assisted Toxicity Evaluation Systems” and used for training and evaluation of the ML model, which achieved a classification performance exceeding 90% in terms of PR, REC, and ACC. Since DILI presents a wide range of histological features annotated in different liver diseases, thus resulting in misdiagnosis, AI could be a valuable support in solving this problem.

Ramot et al. [73] applied a DL algorithm for the segmentation and quantification of fat vacuoles in murine models of NAFLD, including HFD-fed male mice and high-cholesterol-cholate diet-fed mice. The training was conducted using 750 portions of 60 images of size 512 × 512 pixels, while the separate validation set included 75 tiles not overlapping with the training ones. The AI method called AIRA Matrix, which involves selective tiling and subsequent segmentation of individual vacuoles using a CNN encoder–decoder, was applied to the obtained images. The final percentage of vacuoles was then calculated as the ratio of the detected vacuoles to the tissue area. The quantitative output of the model, obtained in real time by connecting the computer to a microscope camera that provides real-time images to the computer, was compared with the semi-quantitative manual microscopic evaluation by an experienced pathologist. This comparison showed a good correlation between the two assessments, with an r value larger than 0.85 demonstrating the effectiveness of automated methods for quantifying steatotic vacuoles. The same authors also proposed a method of accurately quantifying liver fibrosis in mice treated with CCl4 for 8 consecutive weeks [74]. A segmentation task was performed using a U-Net-like encoder–decoder architecture in which the encoding network included modules like those in the Inception architecture, and the decoding network used a dense shortcut connection at each stage. In addition, both blocks used parallel convolutions to improve context aggregation further. Two separate models were used for 10× and 40× magnification images. The entire dataset consisted of 140 field-of-view images of size 3008 × 4112 pixels, of which 80 were acquired from CCl4-treated mice and 60 from control mice. Having too large a size, these images were divided into patches of size 1024 × 1024 pixels, with 25% overlapping in the training and validation sets and no overlapping in the test set. Each image portion was pre-processed by subtracting the mean and dividing each color channel by its respective standard deviation.

Along with CNN, color thresholding in H (hue), S (saturation), and V (value) color space was used as an output. Each image in the training set was normalized between 0 and 1, and threshold values were obtained for the H, S, and V color channels such that more than 90 percent of the collagen regions would be segmented. These values were used during testing. Eventually, the portions of the images were reassembled to obtain the complete original image, of which the outputs of the artificial neural networks (ANNs) and HSV thresholding were combined with the OR operator, followed by a closure operation. The proposed architecture achieved better segmentation performance than most state-of-the-art semantic segmentation models. In addition, an excellent correlation was found by comparing the quantitative assessment of the collagen area performed using the AI algorithm and the semiquantitative assessment performed by a licensed pathologist.

Pischon et al. [75] developed two AI models to assess hepatocellular hypertrophy in livers stained with H&E from rats treated with Phenobarbital for 7 and 14 days. The first model aimed to identify critical tissue areas (centrilobular, midzonal, and periportal), assess the size of cells in these regions, and then calculate the mean cytoplasmic area of hepatocytes. To perform this, once the tissue areas of interest were selected, a classifier was trained to distinguish portal tracts from central veins, and a second DL algorithm was trained to segment sinusoids and hepatocellular nuclei. Finally, the mean hepatocellular cytoplasmic area with its standard deviation was calculated for each animal. When the results of the AI approach were compared with the pathologist classification, a statistically significant correlation between the two evaluations of mean centrilobular cytoplasmic area, relative liver weight, and mean centrilobular hepatocellular cytoplasmic area emerged. In contrast, the second DL model was trained directly to detect hepatocellular hypertrophy compared with normal tissue and quantify hepatocellular hypertrophy by differentiating in liver areas. Also, the results of the DL method significantly correlated with the findings of pathologists. In summary, both approaches, based on U-Net architecture, have proven valuable as tools to support pathologists.

An AI-based solution for preclinical toxicology studies was proposed by Shimazaki et al. [76], in which multiple U-Net-based DL networks were trained to classify and quantify simultaneously different histopathological findings including spontaneous and drug-induced hepatocyte vacuolization, single-cell necrosis, bile duct hyperplasia, hepatocellular hypertrophy, microgranuloma, and extramedullary hematopoiesis on H&E-stained rat livers acquired using WSI systems. Model training was executed using 92 digitized WSIs of livers treated with various compounds during toxicity studies, while the test dataset included 59 WSIs, and the pathologist validation dataset included 255 WSIs. The training images, cropped into small tiles, annotated by data marking experts under the guidance of pathologists, and normalized using the mean and standard deviation of color channels, were used to train the model to detect individual lesions, while the test images were used as feedback to improve the performance of the model from time to time. Finally, using the validation dataset, the performance of the algorithms in terms of quantification and classification of histopathological findings was evaluated. An analysis of WSIs using the algorithm, except for hepatocellular hypertrophy, showed a high correlation with pathologist diagnoses.

A further model for the prediction of damage in DILI was reported by Baek et al. [77]. In particular, the authors applied a Mask R-CNN algorithm to detect and predict various acute liver conditions (including portal triad, necrosis, and inflammation) induced via acetaminophen (APAP) in rats. From the animals, randomly divided into three groups (control group, single-dose APAP group, and repeated-dose APAP group), 201 liver sections were obtained and then stained by H&E and digitized with a 20× objective. Of these, 32 WSIs that were not part of the training dataset were labeled by an experienced pathologist, cropped into patches, classified using the automatic algorithm, and re-aggregated to obtain WSIs again to evaluate the performance of the model. The comparison between automatic classification outputs and pathologist annotations was highly correlated.

Finally, Kim et al. [78] applied AI, specifically the Xception network, to detect and classify liver lesions into normal and fibrosis in mice treated with HFD combined with CCl4 (HFDC) and in a group of HFDC mice treated with elafibranor (ELA) to evaluate the liver damage reversion. Liver tissues were stained with Sirius Red, then 33 WSIs were acquired and digitized. Thirteen WSIs (five from the control group, four from the HFDC group, and four from the HFDC + ELA) were cropped, and each resulting patch was classified as normal or fibrotic livers by a pathologist and then divided into the training, validation, and test sets. The grading accuracy in testing was found to be the highest. A final analysis was performed using the remaining 20 WSIs appropriately cropped to compare the performance of the model with the average grade of three pathologists for each WSI, as well as the researchers’ annotations. Both the correlation between the degree of liver fibrosis predicted using the model and that assigned by the pathologists and the correlation between the ratio of annotated and model-predicted areas of fibrosis was very strong.

All reported studies in animal models strongly support the concept that AI modeling of liver disease coupled with WSI systems could be a valuable tool to support pathologists as a second opinion for practical use in the diagnosis of drug or metabolically induced liver damage.

### 3.4. Studies Conducted on Human Tissues

After an accurate selection, we found 17 articles on the application of AI approaches for the evaluation of tissue images from patients affected by different liver diseases, such as NAFLD, cirrhosis, and hepatocellular carcinoma (HCC). Studies conducted on human liver tissues were distinguished in those using ML methods (Table 3), and those exploited by DL (Table 4).

#### 3.4.1. ML Approaches on Human Tissues

The problem of color information loss related to grayscale image conversion was addressed by Shi et al. [79], who proposed a joint sparse coding-based linear spatial pyramid matching (ScSPM) method (JScSPM) combined with a SVM with a histogram intersection kernel and chi-square kernel for the color classification of histological images of HCC. The JScSPM method was applied both on grayscale or red/green/blue (RGB) images from which the local feature descriptors were extracted using the scale-invariant feature transform algorithm, and the training dictionary was created by sampling and randomly selecting the previously obtained information. The SVM model performed multiclass (well-differentiated, moderately differentiated, and poorly differentiated HCC) and binary (early vs. advanced HCC) classification tasks on these data. Each of the two tasks was repeated five times using the leave-one-out cross-validation strategy, and the results obtained at each repetition were reported as average. Finally, the authors compared the JScSPM method with the original ScSPM and the integrated three-color channels VScSPM method. From this comparison, it has emerged that both JScSPM and VScSPM outperformed ScSPM in all experiments because color information was incorporated in them and that JScSPM also significantly outperformed VScSPM in both classification tasks because the joint dictionary was able to represent not only the color information in each channel but also the intrinsic correlation between different channels.

Liu et al. [80] proposed a new SetSVM ensemble classification approach to build a predictive model adaptable to any set of nuclei in tumor samples. This method solved the set classification problem by jointly optimizing the mapping function and the decision boundary of SVM in a maximum soft-margin problem. The uniqueness of this approach was that it combined set representation learning with classifier training in a single unified cost function to increase the algorithm performance. Among the various tissues on which the model was tested was the hepatic one, for which the diagnostic challenge involved the differentiation of focal nodular hyperplasia from malignant HCC. Liver tissue images were segmented using an unsupervised method, and each nucleus image was normalized at the pixel level (subtraction of the minimum pixel value and division by the max-min distance) and at the position level (elimination of nuclei position variations such as rotation, translation, and coordinate inversions). Different nuclear features, including handcrafted features (e.g., morphological, texture, and wavelet features), as well as features extracted from the latent space of a stacked sparse autoencoder with two layers and transport-based morphometry, were obtained using unsupervised extractions. The SetSVM performance, obtained using the leave-one-out strategy, was high for all types of features, especially for the handcrafted ones. The results also showed that the proposed model, which allowed a visual interpretation of distinctive nuclear features, could be an interpretable and accurate tool.

An automated tool for detecting and quantifying liver fibrosis on digital images of trichrome-stained slides of patients with NAFLD was proposed by Gawrieh et al. [57]. The detection of different types of fibrosis was performed by SVM classifiers with linear kernels trained using morphological features and structural properties of the blue areas extracted from pathologist annotation and correlated with the presence of collagen. All classifiers were subjected to 10-fold cross-validation and showed promising results for determining architectural patterns of fibrosis in liver biopsies from patients with NAFLD. The quantification of fibrosis was performed by measuring the collagen proportional area (CPA), which is calculated as the percentage of the blue pixels area to the total tissue area. Comparisons between the automated measurement of CPA and the scores assigned by pathologists were highly correlated.

Pérez-Sanz et al. [81] developed a CV-based application for the objective, rapid, and automatic quantification of macrovesicular steatosis in histopathological slides of Sudan-stained liver sections from patients with NAFLD. Several windows were manually extracted from the 20 available images, and their pixels were labeled depending on whether they belonged to the region with fat vacuole (label 1) or not (label 0). Then, a vector of six features defined using RGB and CIE L*a*b color spaces was obtained for each labeled pixel. Randomized subsets of pixels were selected to train different models such as k-nearest neighbors (KNN), SVM, RF, naïve Bayes (NB), simple ANN, and ANN with TensorFlow and Keras. Data partitioning, repeated 10 times, was performed using stratified random sampling. KNN and NB were found to be the most precise and fastest algorithms, while SVM and RF were the slowest approaches due to the image size. The comparison between the ML performance and the pathologist manual classification was made by calculating ACC as the ratio of well-classified pixels to the total pixels, SE as the ratio of detected fat vacuole pixels to total fat pixels, and SP as a ratio of detected non-fat vacuole pixels to total non-fat pixels.

To measure the amount of scarring in liver sections stained with PicroSirius Red (PSR), an exploratory study was conducted by Astbury et al. [82]. Twenty explanted cirrhotic livers (four alcoholic hepatopathy, four nonalcoholic fatty hepatopathies, four chronic hepatitis C virus infections, four primary sclerosing cholangitis, and four primary biliary cholangitis) were considered. From each of these, five adjacent sections were cut and subsequently stained at two independent clinical pathology laboratories to study the effect of inter-laboratory staining variation. Six months later, the same staining was performed on other sections from the same subjects to assess intra-laboratory variation. Once the images were acquired, the Waikato Environment for Knowledge Analysis (WEKA) was used to construct the AI-based classifier. Based on pixel features such as intensity mean, minimum, maximum, median, and variance, the multi-class classification involved four classes, including empty space surrounding the extracted portions of tissue, lumen, positive PSR, and tissue. The output was an image segmented into four colors according to the defined classes, and for each class, the number of pixels could be counted. Both individual WEKA classifiers specific to each image set and combined WEKAs that were trained on the tiles drawn from all the colored image sets were developed. Combined training marginally increased individual classifier consistency, so training was repeated to improve classification performance. The results of automatic scar quantification in liver sections showed that inter- and intra-laboratory staining differences had dramatic effects on the classification outputs, in contrast to manual labeling, which was more consistent. Thus, this study demonstrated that the AI method used was more vulnerable to inter- and intra-laboratory staining variation than in humans.

Wan et al. [83] proposed a probabilistic polarization-based discriminative model (P-PDM) for deriving a set of new sigmoid-transformed polarimetry feature parameters (PFP) for the accurate and quantitative characterization of cancer cells in liver cancer tissues. The nodes of such a model were constructed using L0 regularized logistic regression (LR) classifiers and connected using conditional probability and Bayes’s theorem. These classifiers performed better than L1-regularized LR, ANN, and LDA. The pathologists selected 6 ROIs for each liver cancer tissue, resulting in 42 ROIs, from which basic polarimetry parameters (PBPs) were measured using the Muller array microscope that will constitute the P-PDM input data. The best PBPs were selected, which turned out to be the Mueller matrix polar decomposition (MMPD), the Mueller matrix transformation (MMT), the Mueller matrix rotation invariant (MMRI), and a Mueller matrix asymmetry parameter (MMAP). From these data, the model produced sigmoid-transformed PFPs whose shapes depended on the number of nodes used, the edges connecting the nodes, and the probability formulas describing the nodes. Given a pixel from the ROI image, the goal of the P-PDM was to classify the pixels based on its set of PFPs. The P-PDM aimed to determine the probability that a certain pixel belonged to the class of healthy cells or cancer cells and produced two sigmoid-transformed GFPs for characterizing the target microstructures, respectively.

#### 3.4.2. DL Approaches on Human Tissues

Lin et al. [84] fused multiphoton microscopy (MPM) and a deep network based on pre-trained VGG-16 to perform the differentiation of HCC stages. Available HCC specimens were subjected to H&E staining and MPM imaging. From the H&E images, an experienced pathologist diagnosed the HCC grades as well-differentiated (G1), moderately differentiated (G2), and poorly differentiated (G3), also identifying the tumor area on the slice. Only tissues within the tumor region were selected for MPM imaging to allow the classifier to identify the HCC stage of differentiation. The MPM images were converted to matrix pixel data before being entered into the CNN, which had three outputs for the three different grades of HCC. The training was repeated 10 times using the ten-fold cross-validation method that randomly divided the data into 10 groups, of which 9 were used as a training set and 1 as a test set.

To segment highly clustered steatosis droplets, a study proposed a pre-trained Mask R-CNN DL model for the detection of bounding boxes of objects within images and their masks [85]. Liver images were converted to grayscale and binarized using a normalized threshold, and then separate masks of the overlapping steatosis droplets were extracted. Each droplet was characterized by calculating the eccentricity, size, and perimeter in only steatosis candidates with specific ranges of these features (eccentricity, 0.001–6; size, 0.5–4; perimeter, 0.2–1.5). The data were augmented to improve training performance and increase the generalization and robustness of the trained model, then divided into training, validation, and testing sets. Different versions of the ResNet network were used as the backbone of the model, of which ResNet50 returned the best results.

A DL-based integrated region-boundary network, called DeEp LearnINg stEATosis sEgmentation (DELINEATE), was proposed to quantify hepatic steatosis droplets on liver WSIs [87]. DELINEATE network consisted of two sequential modules: a region extraction module based on modified U-Net architecture, called dil-Unet, and a boundary-prediction module for foreground regions and steatosis boundaries based on the combination of a holistically nested neural network (HNN) derived from VGGNet, and a parallel-trained “weighted fusion” layer. This second module also generated an integrated prediction map. The information derived from these two modules was combined to generate the training set for a fully convolutional network with a transposed convolution layer with stride 8 in the final layer (FCN-8), with three output channels representing the probabilities of each input pixel belonging to the background, contour, or region class, respectively. The proposed model provided steatosis prediction at both patch and whole-slide levels. In fact, before the segmentation, each image was normalized to a standard H&E calibration based on the color of the staining and was divided into non-overlapping patches, followed by downstream segmentation. Moreover, image patches were merged with a generic MapReduce-based image analysis framework called MaReI, which generated a steatosis prediction map for each whole tissue component. The metrics F1, REC, Dice index, and Hausdorff distance were used to evaluate the results of steatosis segmentation. Comparison with other models showed superior performance by outperforming the state-of-the-art FCN and DeepLab models. Finally, the calculation of both steatosis pixel percentage (DSP%) and isolated steatosis drop count percentage (DSC%) was performed. Both measurements showed strong correlations with manually confirmed histological and radiological measurements.

Levy et al. [87] presented an end-to-end command-line framework called PathFlowAI for digital clinical image analysis. This system, which included image preprocessing operations, segmentation and classification using DL, and pattern interpretation, was applicable at both patch and whole-slide levels. The preprocessing phase consisted of reading and storing the user-supplied data (images and their annotation masks), dividing them into patches, and building an SQL database containing some annotation information. After data storage, the images were analyzed using DL, and the user could choose from several classifications (VGG, ResNet, Inception, EfficientNet, and AlexNet) and segmentation (UNET, FCN, Fast-SCNN, and DeepLab) models, most of which were pre-trained on ImageNet. Finally, PathFlowAI provided both a way to visualize classification and segmentation outputs and a tool to understand model predictions through an interpretation analysis using the Uniform Manifold Approximation and Projection for Dimension Reduction (UMAP) embeddings and SHapley Additive exPlanations (SHAP) method. The utility of the proposed tool was demonstrated by analyzing 23 WSIs of liver biopsies for steatohepatitis assessment using both classification (patch level) and segmentation (pixel level). A U-Net model was used to segment portal tissue from parenchyma and background on fine patches of 256 × 256 pixels, with high average SE. Portal tissue classification, performed on patches of 512 × 512 pixels using a ResNet34 architecture, demonstrated agreement with the assessment by a pathologist. The obtained results suggested that the proposed workflow represented a time- and performance-efficient clinical application tool. The same authors also conducted a large-scale internal validation study of the feasibility of generative adversarial networks (GANs) for converting digital WSIs from H&E to trichrome staining [88]. Twenty H&E/trichrome WSI pairs of liver tissues from nonalcoholic steatohepatitis (NASH) patients, covering a representative sample of fibrosis stages, were divided into smaller sub-images and used for training the CycleGAN model that generated virtual staining. To evaluate the visual quality of the model output, multiple Turing tests were conducted with the help of four pathologists who, in 9 of the 12 tests, were unable to distinguish virtual from real images. The same pathologists inspected the virtual and real images separately for each slide using both the Automated Slide Analysis Platform (ASAP) and the OpenSeadragon viewer and classified them independently. On a large-scale in-house validation cohort, a strong correlation in staging between real and virtual stains assessed by pathologists was quantitatively demonstrated.

The problem of cleaning annotations of cancerous regions in the WSIs was addressed by Wang et al. [89], who proposed a framework called label cleaning–multiple-instance learning (LC-MIL) to refine coarse labels. LC-MIL is a multiple-instance learning (MIL) framework for modeling imperfections as patch-level noise, identifying erroneous annotations, and correcting them. The authors proposed two variants, LC-MIL-atten and LC-MIL-miNet, which shared the same MIL structure based on the pre-trained VGGNet16 architecture but differed in the predictor. Coarse annotations (simulations, S) were generated in different ways by uniformly flipping positive and negative samples as different noise frequencies between 0 and 0.5 (S-I), by purposely omitting small lesions (S-II), and by asking two experienced pathologists to annotate each WSI in only 30 s (S-III). In all cases, the LC-MIL method significantly refined the simulation even when learning occurs on only one WSI, thus easing the pathologist’s work.

Cinar et al. [91] proposed a new classification method of HCC using a customized imaging system and a 3D CNN. The acquisition system, which integrated a hyperspectral camera and an optical microscope with a 3D-printed motorized stepper, captured hyperspectral images (HSI) of healthy and cancerous tissue samples from a liver microarray slide. The images captured from each sample were divided into smaller patches before being used for AI model training. A 3D CNN was chosen because 3D kernels allowed the network to extract spatial and spectral voxel information with a compact approach. The cost function used was a focal loss function, which helped overcome the class imbalance problem and emphasized the hard-to-learn examples by increasing the generalization of the model without causing overfitting. This cost function involved two hyperparameters, alpha and gamma, which were optimized during the training phase of the model. This approach demonstrated both the superiority of 3D convolutions over 2D convolutions in terms of classification ACC and the superiority of HSI data over RGB data for liver cancer tissue classification.

A panel of multiplex immunofluorescence for different prognostic markers (i.e., CD3 as pan T lymphocytes, CD4 for T helper cells, CD8 for cytotoxic T lymphocytes, FoxP3 for Treg lymphocytes, and PD-L1) combined with DAPI to evaluate which analytical approach was best suited to combine morphologic and immunohistochemical data into a cancer score to identify the area of cancer that best matched an independent pathologist assignment [92]. Individual features were extracted from each cell and used for calculating a cancer score with four different approaches: a correlation-based individual cellular feature selection, a MANOVA-based feature selection, a multilayer perceptron (MLP), and a U-Net network. With an average ACC of 75%, the U-Net network was the best model to identify the cancer area.

Zhan et al. [93] proposed a tool for histologically grading liver fibrosis by combining MPM and DL techniques in patients with metabolic dysfunction-associated fatty liver disease (MAFLD). Such a tool called the automated liver fibrosis grading network (AutoFibroNet) exploited a four-layer MLP network to combine clinical features of patients, collagen features manually extracted, and features automatically extracted from a CNN (5D features). For the selection of the DL model to be used, the pre-trained models ResNet34, MobileNetV3, and VGG16 were tested. VGG16 resulted in the best in terms of both ACC and loss rate. AutoFibroNet was tested on two independent validation cohorts, demonstrating how the combination of DL approaches and SHG/TPEF was a potentially useful tool for the assessment of liver fibrosis.

Interestingly, Wei et al. [94] proposed a cross-modality translation method based on DL and polarization imaging to help pathologists analyze the properties of histological specimens. H&E-stained and immunohistochemistry-stained liver tissues from different etiologies, as well as other tissues, were used to demonstrate the effectiveness of a method that used snapshot Stokes polarimetric images as inputs for a CycleGAN network for the generation of a virtual bright-field image resembling closely the real one. The obtained results, measured using the structural similarity index (SSIM), root-mean-square error (RMSE), Jensen–Shannon divergence (JSD), and earth mover’s distance (EMD) for different polarization states (linear at 45 and 135 degrees; right circular (R) and left circular (L); elliptical E1 and E2), showed that the proposed method not only saved time, labor, and cost but also avoided the errors caused by light intensity instability and image misregistration in the MM microscopy.

Finally, Becker et al. [95] proposed a screening of pathological collagen-rich regions in ex vivo livers from control subjects: patients with HCC and liver cirrhosis or patients with liver cirrhosis associated with biliary atresia. The authors reported that marker-independent Raman microspectroscopy images per se analyzed using DL and ML approaches were unable to distinguish fibrotic from non-fibrotic tissue, while the implementation of Raman images with information obtained via Collagen 1 staining using immunofluorescence could be able to discern the accumulation of this component in fibrotic compared to control livers. The study did not provide performance obtained using only liver imaging, but by composite images from different tissues; thus, it was not included in Table 4.

## 4. Discussion

### 4.1. State-of-the-Art

Applications of CV in medicine are becoming increasingly important, especially in the field of imaging [96]. Histopathological images are the gold standard for evaluating certain types of diseases. The analysis of those images requires time and resources, and it is very challenging even for experienced pathologists [97], who are called to observe the slides using a microscope, thus leading to inter-observer and intra-observer variability in the final annotations. These reasons have led to the increased demand for analysis using CAD systems, which require some steps for the inspection of histopathological images, including the division of WSI’s into patches of reduced size (with or without overlap), the subsequent steps of pre-processing, segmentation, features extraction, and application of ML or DL approaches. Of note, a recent article provided a comprehensive, systematic, and updated discussion of the literature evidence on the application of DL approaches on WSIs obtained via liver histopathology [98]. In particular, the authors evaluated the selected articles by highlighting their performance and bias through a useful tool known as the Quality Assessment of Diagnostic Accuracy Studies version 2 (QUADAS-2). Even though the use of QUADAS-2 is encouraged by current PRISMA guidelines [67], this tool is not completely adaptable to evaluate AI-centered diagnostic test accuracy, which requires the completion of a QUADAS-AI tool [99]. For these reasons, the present systematic review was limited to summarizing ten-year evidence of applications of the two main branches of AI (i.e., ML and DL) for the evaluation of histological images of hepatic tissues from patients affected by different liver diseases from the perspective of DP without using QUADAS-2.

Our search in the electronic databases PubMed, SCOPUS, and Embase resulted in the selection of 28 articles focused on the topic of the present review and reporting the results obtained in terms of the performance of the developed AI models for DP in liver diseases. Based on the data collected from the selected articles, we conducted a qualitative analysis and highlighted the main critical issues found in DP.

### 4.2. Qualitative Analysis

**Figure 4:**
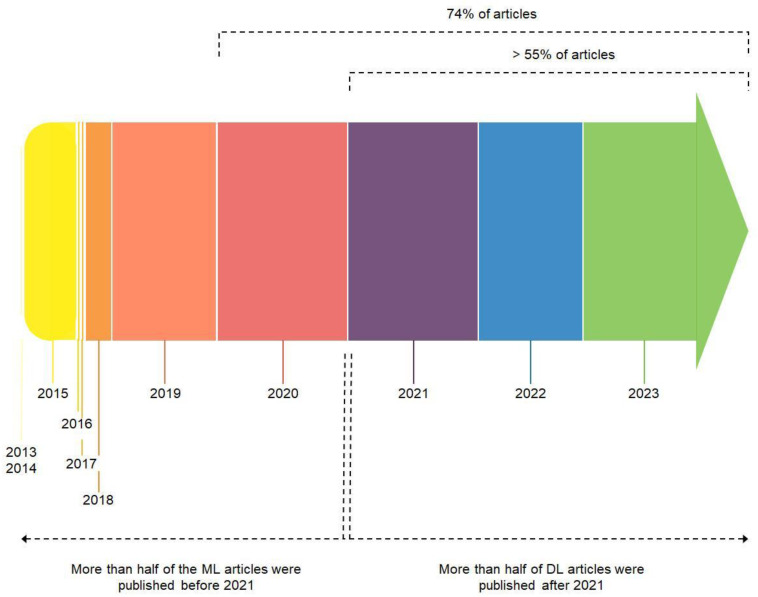
Timeline diagram of articles published from January 2013 to November 2023 on the topic of applications of AI to DP. The size of each time block is proportional to the number of articles published in the corresponding year.

Although the search included publications since 2013, the first study that reported AI approaches applied to DP was published in 2015, and more than half of the selected articles were published between 2021 and 2023. Interestingly, over 88% of the selected articles were published within the past five years. In addition, over half of the ML articles were published by 2021, and over half were published from January 2021 to November 2023. A timeline diagram that assigns the size of the various blocks (each related to a year of publication) based on the percentage of articles published is reported in Figure 4.

**Figure 5:**
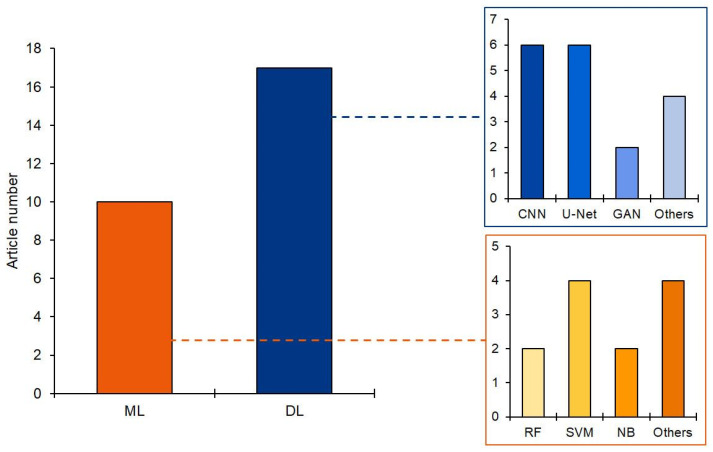
Number of articles using ML or DL approaches and analysis of the most frequently used models.

The analysis also showed that the most widely used ML classifier was the SVM model, while CNN (custom, pre-trained, or region-based) and U-Net were the most common in DL approaches, where image classification and segmentation were prerequisites for feature extraction. Moreover, among the many solutions identified, DL-based approaches were the most frequently used, accounting for 63% of the articles (Figure 5). Indeed, DL and CV techniques hold great promise for improving the accuracy of liver disease identification and classification.

**Figure 6:**
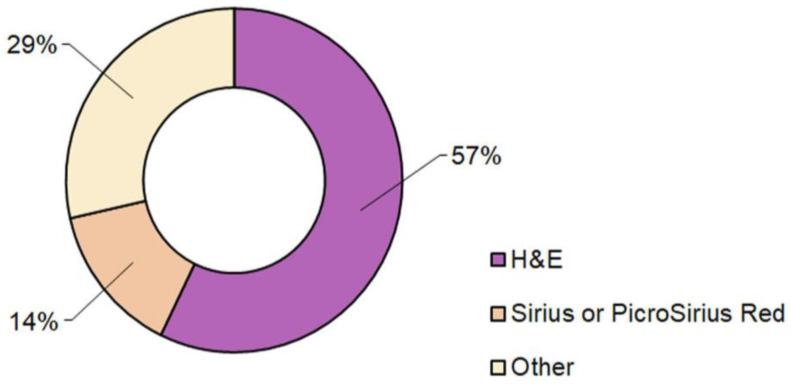
Pie chart of the percentage distribution of staining used for histopathological imaging.

In analyzing the various articles reported in this review, we also noticed the variability in the type of staining of histopathological images. Although H&E images were used in more than half of the studies, a small percentage used Sirius or PicroSirius Red staining, and nearly 30% used other staining techniques, thus highlighting that the choice of type of staining was strongly related to the goal of the study (e.g., steatosis, steatohepatitis, fibrosis, neoplastic lesions, or HCC). The distribution in the percentage of staining types used for histopathological imaging in the selected articles is shown in the pie chart in Figure 6.

### 4.3. Generalizability

An important aspect to consider when applying AI algorithms in real-world settings such as medicine is their ability to generalize, that is, to show high classification, segmentation, or prediction performance on datasets different from the one used in the training. Among the articles selected in this review, only four discussed a generalized AI model that exhibited good performance in different tissues, thus increasing the robustness of the proposed approach. The results obtained, which include only human tissues, are shown in Table 5. Comparing these results with those of the related authors summarized in Table 3 and Table 4, the stability of performance obtained using the same algorithms applied to different tissues can be seen. This shows that the proposed networks are robust to variations in input and task, a quality that is important in applications of AI methods and without which CAD systems can hardly be integrated into clinical practice.

### 4.4. Overall Bias in the Selected Articles

Complexly, our findings revealed that even if ML and DL are continuously being updated, the use of DL algorithms is growing rapidly because these approaches are more powerful in solving complex medical questions related to large amounts of unlabeled or unstructured data. Moreover, the proliferation of high-quality labeled big data, optimization algorithms, and the development of specialized software also largely contribute to the use of DL algorithms to evaluate medical images [100]. However, even though the performance of the selected studies is good, some of them use a small number of images for DL algorithm training, thus making acquiring the ground-truth response labels associated with the input features very difficult.

### 4.5. Limits of DP

Despite promising results, the use of DP for imaging pre-processing and segmentation and its combination with AI approaches to extract features that could offer a software solution for use in clinical settings still present some limitations [101].

Currently, WSIs can serve as an effective surrogate for traditional microscopy-based pathology workflow. Indeed, WSI systems may provide a complete representation of a glass microscope slide at any magnification. However, WSI requires dedicated equipment and several high-cost infrastructures, including scanners, workstations, and substantial network bandwidth, to handle large file sizes. Another problem may be represented by discrepancies between WSI and glass slides, which include detecting microorganisms and identifying mitosis and specific nuclear morphologies or characteristics in slides obtained from tumor specimens [101]. A further relevant limitation of WSI scanners is associated with their inability, in most cases, to acquire multiple planes in tissue samples. Several high-throughput scanners have recently been introduced, but their use is limited by the requirement of a large amount of time for acquisition and large storage facilities for data, but most importantly, high cost.

Further aspects that should be solved in a DP workflow combined with AI approaches for diagnostic goals include the improvement of the appropriateness of image labeling before segmentation and feature extraction, the increase in quality and quantity of image datasets for training, validation, and testing, and the development of strategies and tools for accurate cross-validation and interpretability of AI models.

In DP, the process of generating labels associated with histopathology images is laborious or expensive, but it also underlies any supervised learning method. Most currently available DP images involve case-level annotation (disease name) and, in some cases, such as those of cancer, even if the entire image is labeled as cancerous, it is possible that in it, cancer covers only a small part of the slide, while for the most part, the tissue is not cancerous [102]. In addition, the relative position of the cancerous area within the WSI can vary greatly from case to case. This “weak annotation” problem significantly reduces the performance of AI models. Furthermore, labeling is a process that requires histopathological expertise and can only be performed on relatively small datasets. In the absence of labeled data, one can consider using unsupervised approaches that can extract relevant information by studying the models in unlabeled training data [103].

A further problem encountered in pathological image analysis using AI is the lack of a sufficient amount of high-quality data, without which AI algorithms may not be able to learn fundamental patterns or relationships to make accurate predictions and diagnoses, and the lack of diversity in medical data that could lead to biased algorithms that may not perform well on data from different populations or demographics. Because CV application requires a large dataset with detailed annotations for both the training and validation phases, there is a need to increase the collection and sharing of high-quality medical data through collaborations between healthcare professionals, researchers, and technology companies, as well as through the development of data sharing platforms and standards that ensure privacy and security. Recent studies have shown that despite the use of meticulously labeled and pixel-wise data, the performance of AI models decreases by 20 percent when trained on data sets that are too small and tested on independent data sets. To obtain models with a high level of generalizability, it is necessary to make sure that the training data includes a large and representative sample of the biological and morphological variability of the disease, as well as the technical variability introduced in the preanalytical and analytical processes in histopathology and the imaging acquisition process [104]. Several public datasets containing hand-annotated histopathology images exist in the field of DP [105,106,107]. However, these datasets could be useful if the purpose of analysis, slide conditions (such as staining), and image conditions (such as magnification level and image resolution) were similar [108]. The generalization error of AI models could be greatly reduced if training and tuning were performed on large and diverse datasets. Indeed, efforts should be made to increase the diversity of medical data to avoid bias in AI algorithms and to ensure that AI technologies are developed in a way that benefits all patients.

Related to the latter issue, another problem that emerged from the analysis is the lack of external cross-validation, especially in cancer classification [109]. Without this, it is not possible to determine whether AI techniques are truly ready to be introduced into routine clinical practice.

Moreover, to integrate these algorithms into healthcare systems, the problems of reliability and interpretability must be addressed [101,110]. Indeed, although ANNs excel at many complex tasks, they are often considered “black boxes” [42] because it is difficult to interpret the model (usually with millions of parameters), retrieve biological information, and obtain meaningful information [111]. Also, in the field of histopathology, it is important to apply explainable AI (XAI) techniques that can help improve the comprehensibility of AI solutions.

### 4.6. Present Review Limitation

The present systematic review suffers from the generalized bias associated with search strategies, inclusion and exclusion criteria, and the limited number of used databases. In general, language and timeline restrictions increase the risk of bias because they may hinder relevant articles with a high performance. To conclude, we believe that these limitations may have a minor weight on analyzing a quite young field, such as AI.

### 4.7. Future Directions of AI Approaches in Liver Diseases

Histological evaluation remains a key tool for the evaluation of types and entities of liver damage in patients affected by different liver diseases, including NAFLD/MAFLD, DILI, alcoholic liver disease, cholestatic diseases, metabolic/inherited disorders, and HCC [112]. In this context, DP coupled with AI-based feature extraction has shown recent exciting results for histopathological diagnoses, particularly in the field of NAFLD, where this workflow may be used to automatically detect, localize, and quantify histological features that could be classified for severity using AI-generated scores. In particular, Sanyal et al. [113], in a recent perspective, discussed key positive points and gaps in knowledge for the use of AI-supervised and unsupervised approaches for the detection of NASH-related features and staging of fibrosis in NAFLD/MAFLD.

Up to date, AI tools are not too distant from being included in clinical practice in the field of DP. Nevertheless, uncertainties as to the current extent of clinically relevant benefits remain in the face of reliability in the use of combined WSIs and AI approaches, which encompasses image quality (e.g., resolution, clarity, and consistency of images), accuracy of the AI approach, standardization of protocols for image acquisition/storage/analysis, validation and verification, and by pathologists.

Overall, DL techniques have gained a lot of traction among researchers because of their optimized performance. However, in the future, it is necessary to improve DL approaches with techniques for noise reduction and XAI. Large amounts of data should be collected and well-stored to increase accuracy, and the model’s capacity to handle big datasets should be enhanced. Moreover, when introducing AI into medicine, ethical aspects related to cybersecurity and data integrity in the workflow, protection of patient privacy, and ensuring equitable access to AI-based health care must also be considered [114].

## 5. Conclusions

As a whole, the studies reported in this review article have shown that a workflow that combines DP and AI applied to liver disease can become a useful tool to support medical research and clinical decision making in the future, leading to better patient outcomes and more efficient healthcare.

## Figures and Tables

**Table 1:** Overview of cited works using images of animal tissues and ML models (results have been rounded). The symbol “-” indicates that no information is provided on a particular operation.

Tool Used	Study Aim	Model of Animals	Data Augmentation	Results	Author and Year
RF	Fat droplet identification for rapid and accurate quantification of hepatic steatosis	NAFLD in rats with methionine-choline-deficient plus high-fat diet	-	SE SFD: 0.91 ± 0.02; SE CFD: 0.90 ± 0.02; SP: 0.92 ± 0.01; ACC: 0.91 ± 0.01	Homeyer et al., 2015 [68]
K-means and BoF framework	Analysis of fibrosis states via the quantification of spectral–spatial features	Carbon Tetrachloride-induced hepatic fibrosis in mice	-	ACC: 0.90–0.95	Saitou et al., 2018 [69]
LDA	Analysis of sepsis-induced liver damage	Polymicrobial abdominal infection in mice	-	SHG AUC: 0.49; TPEF AUC: 0.83; CARS AUC: 0.93; SHG/TPEF/CARS AUC: 0.85	Yarbakht et al., 2019 [70]
Classification and Regression Trees	Analysis of steatosis and fibrosis states using label-free microscopy	Mice model of diet-induced NAFLD	-	Steatosis AUC: [0.81–1] Fibrosis AUC: [0.73–1]	Wang et al., 2023 [71]

RF: random forest, NAFLD: non-alcoholic fatty liver disease, SE: sensitivity, SFD: separated fat drops, CFD: clustered fat drops, SP: specificity, ACC: accuracy, BoF: bag-of-features, LDA: linear discriminant analysis, SHG: second harmonic generation, TPEF: two-photon excitation fluorescence, CARS: coherent anti-Stokes Raman scattering (CARS).

**Table 2:** Overview of cited works using images of animal tissues and DL models (results have been rounded). The symbol “-” indicates that no information is provided on a particular operation.

Tool Used	Study Aim	Model of Animals	Data Augmentation	Results	Author and Year
Google’s AutoML Visionbeta	Detection and classification of DILI	DILI induced with different drugs in rats	-	PR: 0.93; REC: 0.93; ACC: 0.93	Puri 2020 [72]
AIRA Matrix	Quantification of fat vacuoles	NAFLD in mice by a high-fat diet or high-cholesterol-cholate diet	-	r = 0.87; *p* < 0.001	Ramot et al., 2020 [73]
U-Net-like custom encoder–decoder architecture	Quantification of fibrosis	Carbon tetrachloride-induced hepatic fibrosis	Random affine transformations and color variations on each training tile	IoU: 0.80; F1: 0.98	Ramot et al., 2021 [74]
U-Net22	Evaluation of hepatocellular hypertrophy	Hepatocellular hypertrophy induced via treatment with Phenobarbital for 7 and 14 days in rats	Rotation, flipping of labeled tissue regions, and changes in brightness and contrast	Model 1—study 1: r = 0.874; study 2: r = 0.705. Model 2—study 2: r = 0.80	Pischon et al., 2021 [75]
U-Net	Simultaneous classification and quantification of different histopathological findings	Hepatocellular damage induced via treatment with different drugs in rats	Chromatic (changes in saturation and hue) and geometric (rotation at multiple 90° angles and horizontal/vertical flips) increments	Lesion detection task—AUC: 0.89; REC: 0.85; SP: 0.82; PR: 0.67; ACC: 0.83; F1: 0.73	Shimazaki et al., 2022 [76]
Mask R-CNN	DILI prediction	DILI induced via treatment with acetaminophen in rats	Reversing, rotating, and changing brightness (8 repetitions)	Portal triad Av PR: 0.95; R^2^: 0.94. Necrosis Av PR: 1.00; R^2^: 0.955. Inflammation Av PR: 0.96. Infiltration Av PR: 0.94. Total Av PR: 0.96	Baek et al., 2022 [77]
Xception	To determine whether an AI algorithm can help the classification of liver fibrosis lesions	Liver lesions induced via treatment with HFD combined with CCl4 (HFDC) in mice and treatment with elafibranor in HFDC mice	-	ACC test: 1.00. Single slide PR: 0.85; REC: 0.96; F1: 0.90. Model vs. pathologists: r = 0.91. Model vs. annotations: r = 0.83	Kim et al., 2023 [78]

DILI: drug-induced liver injury, PR: precision, REC: recall, ACC: accuracy, NAFLD: non-alcoholic fatty liver disease, r: Spearman’s correlation coefficient, IoU: intersection over union, F1: F1 score, AUC: area under receiver operating characteristic (ROC) curve, SP: specificity, R^2^: coefficient of determination, AI: artificial intelligence, HFD: high-fat diet, HFDC: HFD + CCl4.

**Table 3:** Overview of cited works using images of human tissues and ML models (results have been rounded). The symbol “-” indicates that no information is provided on a particular operation.

Tool Used	Study Aim	Disease	Data Augmentation	Results	Author and Year
JScSPM method + SVM	Demonstrate the loss of color information related to the conversion of medical images to grayscale	HCC	-	Multiclass HIK-SVM ACC: 0.92 ± 0.0129; SE: 0.92 ± 0.0134; SP: 0.96 ± 0.0068. Multiclass CSK-SVM ACC: 0.92 ± 0.0136; SE: 0.91 ± 0.0134; SP: 0.96 ± 0.0067. Binary HIK-SVM ACC: 0.96 ± 0.083; SE: 0.9 ± 0.0213; SP: 0.99 ± 0.0122; AUC: 0.981. Binary CSK-SVM ACC: 0.96 ± 0.0166; SE: 0.89 ± 0.0261; SP: 0.996 ± 0.0099; AUC: 0.98	Shi et al., 2015 [79]
SetSVM	Differentiation of malignant HCC from focal nodular hyperplasia	HCC	-	(1) ACC: 0.91; PR: 0.93; REC: 0.82; AUC: 0.93. (2) ACC: 0.85; PR: 0.86; REC: 0.71; AUC: 0.91. (3) ACC: 0.86; PR: 0.92; REC: 0.71; AUC: 0.87.	Liu et al., 2019 [80]
SVM with linear kernels	Detection and quantification of liver fibrosis	NAFLD	-	CPA R^2^: 0.67 and 0.86. Detection task—Av PR: 0.81; Av REC: 0.85; Av AUC: 0.88	Gawrieh et al., 2020 [81]
KNN, SVM, RF, NB, simple ANN, and ANNs with TensorFlow and Keras	Determination of macrovesicular steatosis	NAFLD	-	KNN ACC: 0.996; SE: 0.84; SP: 0.999; PR: 0.96. SVM ACC: 0.996; SE: 0.96; SP: 0.997; PR: 0.89. RF ACC: 0.996; SE: 0.96; SP: 0.997; PR: 0.89. NB ACC: 0.997; SE: 0.91; SP: 0.999; PR: 0.97. ANN ACC: 0.997; SE: 0.96; SP: 0.998; PR: 0.91. Keras ACC: 0.995; SE: 0.97; SP: 0.996; PR: 0.96	Pérez-Sanz et al., 2021 [82]
WEKA	Quantification of scarring in liver sections and study of the effect of staining variation	Cirrhosis from different etiologies	-	WEKA individuals r*_inter_* = 0.24, r*_intra_* = 0.24; WEKA combined (1) r*_inter_* = 0.29, r*_intra_* = 0.31; WEKA combined (2) r*_inter_* = 0.37, r*_intra_* = 0.53	Astbury et al., 2021 [83]
L0 regularized LR	Quantitative characterization of cancer cells	Liver cancer	-	P-PDM ACC: 0.85; REC: 0.88; PR: 0.88	Wan et al., 2022 [84]

JScSPM: joint sparse coding-based SPM method, SVM: support vector machine, HCC: hepatocellular carcinoma, HIK: histogram intersection kernel, ACC: accuracy, SE: sensitivity, SP: specificity, CSK: chi-square kernel, AUC: area under receiver operating characteristic (ROC) curve, PR: precision, REC: recall, NAFLD: nonalcoholic fatty liver disease, CPA: collagen proportional area, R^2^: coefficient of determination, KNN: k-nearest neighbors, RF: random forest, NB: naïve Bayes, ANN: artificial neural network, WEKA: Waikato Environment for Knowledge Analysis, LR: linear regression, P-PDM: probabilistic polarization-based discriminative model.

**Table 4:** Overview of cited works using images of human tissues and DL models (results have been rounded). The symbol “-” indicates that no information is provided on a particular operation.

Tool Used	Study Aim	Disease	Data Augmentation	Results	Author and Year
Pre-trained VGG-16	Classification of HCC differentiation	HCC	Horizontal or vertical rotation and flipping	G1 vs. G2G3 AUC: 0.92; Av ACC: 0.94 (0.913–0.968). G2 vs. G1G3 AUC: 0.89; Av ACC: 0.86 (0.807–0.910). G3 vs. G1G2 AUC: 0.91; Av ACC: 0.81 (0.756–0.868)	Lin et al., 2019 [85]
Pre-trained Mask R-CNN with a ResNet50 backbone	Steatosis segmentation	NAFLD	Random affine transformation, random flipping, and Gaussian blurring	Av PR: 0.76; REC: 0.61; F1: 0.66; Jaccard index: 0.77	Guo et al., 2019 [86]
DeEp LearnINg steatosis sEgmentation (DELINEATE) architecture: dil-U-Net + HNN + FCN-8s	Quantification of hepatic steatosis drops	NAFLD	Region module: horizontal flip, vertical flip, rotation in 4-degree angles, and re-scaling by 0.5 scales. Boundary module: rotation and flipping at 16 different angles	Identification task PR: 0.98 ± 0.01; REC: 0.91 ± 0.06; F1: 0.94 ± 0.03; Object wise Dice Index: 0.9492; Object wise Hausdorff Distance: 3.4591	Roy et al., 2020 [87]
U-Net and ResNet34	Assessment of steatohepatitis pattern	Steatohepatitis from undescribed etiology	-	Av SE: 0.71; Av AUC: 0.78	Levy et al., 2020 [88]
CycleGAN	Conversion of staining of digital WSIs from H&E to trichrome using a GAN	NASH	-	r = 0.86; 95% CI: 0.84–0.88	Levy et al., 2021 [89]
LC-MIL	Refinement of coarse annotation of cancerous regions in WSIs	HCC	-	LC-MIL-atten F1—S-I: 0.91 ± 0.098; S-II: 0.83 ± 0.128; S-III: 0.83 ± 0.163. LC-MIL-miNet F1—S-I: 0.88 ± 0.156; S-II: 0.84 ± 0.119; S-III: 0.83 ± 0.167	Wang et al., 2022 [90]
3D CNN	Classification of HCC	HCC	-	Gamma = 2, and alpha = 0.5: ACC: 0.97; PR: 0.999; REC: 0.97; F1: 0.98; MCC: 0.86	Cinar et al., 2023 [91]
U-Net	Identification of cancer area by cancer score	HCC	-	ACC: 0.75	Dievernich et al., 2023 [92]
AutoFibroNet	Classification of fibrosis grades in MAFLD patients	NAFLD/MAFLD	-	AUC G0,G1,G2,G3/4—first cohort: 0.99, 0.83, 0.80, 0.90; second cohort: 1.00, 0.83, 0.80, 0.94	Zhan et al., 2023 [93]
CycleGAN	Development of a system to support the analysis of the properties of histological specimens	Liver samples from patients of undescribed etiology	-	Stokes images: 45 degrees—SSIM: 0.694; RMSE: 0.099; JSD: 0.181; EMD: 8.935. 135 degrees—SSIM: 0.710; RMSE: 0.104; JSD: 0.203; EMD: 11.515. R—SSIM: 0.732; RMSE: 0.099; JSD: 0.178; EMD: 9.353. L—SSIM: 0.713; RMSE: 0.101; JSD: 0.186; EMD: 9.077. E1—SSIM: 0.706; RMSE: 0.102; JSD: 0.209; EMD: 11.093. E2—SSIM: 0.7518; RMSE: 0.093; JSD: 0.178; EMD: 8.467	Wei et al., 2023 [94]

HCC: hepatocellular carcinoma, G1: well-differentiated HCC, G2: moderately differentiated HCC, G3: poorly differentiated HCC, AUC: area under receiver operating characteristic (ROC) curve, ACC: accuracy, CNN: convolutional neural network, NAFLD: nonalcoholic fatty liver disease, PR: precision, REC: recall, F1: F1 score, HNN: holistically nested neural network, FCN: fully convolutional network, SE: sensitivity, WSIs: whole slide images, H&E: hematoxylin and eosin, GAN: generative adversarial network, NASH: nonalcoholic steatohepatitis, r: Spearman’s correlation coefficient, LC-MIL: label cleaning–multiple instance learning, MCC: Matthews’ correlation coefficient, MAFLD: metabolic dysfunction-associated fatty liver disease, SSIM: structural similarity index, RMSE: root-mean-square error, JSD: Jensen–Shannon divergence, EMD: earth mover’s distance.

**Table 5:** Overview of the articles with generalized AI models (results have been rounded).

Approach	Tissue	Results	Author and Year
SetSVM (ML)	Thyroid tissue sections stained with the Feulgen technique, and melanoma tissue sections stained using H&E	Thyroid cancer: Differentiation of follicular adenoma of the thyroid (FA) from nodular goiter (NG) [AUC: 0.83, 0.84, 0.80]; differentiation of follicular variant of papillary thyroid carcinoma (FVPC) from NG [AUC: 0.87, 0.91, 0.92]. Melanoma: Differentiation of malignant melanoma from dysplastic nevi [AUC: 0.75, 0.75, 0.78].	Liu et al., 2019 [78]
L0 regularized LR (ML)	Breast cancer pathological tissues stained with H&E	P-PDM ACC: 0.87; REC: 0.87; PR: 0.88.	Wan et al., 2022 [82]
LC-MIL (DL)	Breast cancer lymph node metastasis tissues stained with H&E and colorectal cancer tissues stained with H&E	Breast cancer: LC-MIL-atten F1—S-I: 0.88 ± 0.068; S-II: 0.85 ± 0.084; S-III: 0.85 ± 0.096. LC-MIL-miNet F1—S-I: 0.87 ± 0.104; S-II: 0.84 ± 0.091; S-III: 0.84 ± 0.096. Colorectal cancer: LC-MIL-atten F1—S-I: 0.82 ± 0.167; S-II: 0.72 ± 0.138; S-III: 0.86 ± 0.077. LC-MIL-miNet F1—S-I: 0.81 ± 0.181; S-II: 0.79 ± 0.106; S-III: 0.87 ± 0.081.	Wang et al., 2022 [88]
CycleGAN (DL)	Breast tissues pathological slices with H&E staining, and lung tissues with two types of immunohistochemistry staining, i.e., thyroid transcription factor-1 and Ki-67	Breast cancer: Stokes images: 45 degrees—SSIM: 0.727; RMSE: 0.130; JSD: 0.200; EMD: 9.761. 135 degrees—SSIM: 0.742; RMSE: 0.126; JSD: 0.191; EMD: 9.296. R—SSIM: 0.742; RMSE: 0.139; JSD: 0.202; EMD: 11.955. L—SSIM: 0.752; RMSE: 0.134; JSD: 0.226; EMD: 11.711. E1—SSIM: 0.760; RMSE: 0.126; JSD: 0.202; EMD: 9.796. E2—SSIM: 0.755; RMSE: 0.124; JSD: 0.192; EMD: 9.460. Lung cancer: Ki-67: Stokes images: 45 degrees—SSIM: 0.929; RMSE: 0.078; JSD: 0.237; EMD: 7.520. 135 degrees—SSIM: 0.923; RMSE: 0.075; JSD: 0.229; EMD: 6.870. R—SSIM: 0.917; RMSE: 0.083; JSD: 0.248; EMD: 7.914. L—SSIM: 0.911; RMSE: 0.081; JSD: 0.237; EMD: 7.322. E1—SSIM: 0.920; RMSE: 0.082; JSD: 0.240; EMD: 7.738. E2—SSIM: 0.908; RMSE: 0.093; JSD: 0.23; EMD: 7.693. TTF-1: Stokes images: 45 degrees—SSIM: 0.915; RMSE: 0.087; JSD: 0.246; EMD: 6.698. 135 degrees—SSIM: 0.904; RMSE: 0.101; JSD: 0.277; EMD: 8.384. R—SSIM: 0.917; RMSE: 0.092; JSD: 0.267; EMD: 7.360. L—SSIM: 0.925; RMSE: 0.092; JSD: 0.261; EMD: 7.519. E1—SSIM: 0.9214; RMSE: 0.097; JSD: 0.263; EMD: 7.998. E2—SSIM: 0.914; RMSE: 0.091; JSD: 0.253; EMD: 7.194.	Wei et al., 2023 [92]

SVM: support vector machine, ML: machine learning, H&E: hematoxylin and eosin, AUC: area under receiver operating characteristic (ROC) curve, LR: linear regression, P-PDM: probabilistic polarization-based discriminative model, ACC: accuracy, REC: recall, PR: precision, LC-MIL: label cleaning–multiple-instance learning, DL: deep learning, F1: F1 score, GAN: generative adversarial network, SSIM: structural similarity index, RMSE: root-mean-square error, JSD: Jensen–Shannon divergence, EMD: earth mover’s distance.

## Data Availability

Not applicable.
